# Cryptosporidiosis: From Prevention to Treatment, a Narrative Review

**DOI:** 10.3390/microorganisms10122456

**Published:** 2022-12-13

**Authors:** Yosra A. Helmy, Hafez M. Hafez

**Affiliations:** 1 Department of Veterinary Science, College of Agriculture, Food, and Environment, University of Kentucky, Lexington, KY 40546, USA; 2 Institute of Poultry Diseases, Freie Universität Berlin, 12309 Berlin, Germany

**Keywords:** cryptosporidiosis, one health, poultry, vaccines, epidemiology, waterborne pathogen, foodborne pathogen, outbreaks

## Abstract

Cryptosporidiosis is a water- and food-borne zoonotic disease caused by the protozoon parasite of the genus *Cryptosporidium. C. hominis* and *C. parvum* are the main two species causing infections in humans and animals. The disease can be transmitted by the fecal–oral route as well as the respiratory route. The infective stage (sporulated oocysts) is resistant to different disinfectants including chlorine. Currently, no effective therapeutic drugs or vaccines are available to treat and control *Cryptosporidium* infection. To prevent cryptosporidiosis in humans and animals, we need to understand better how the disease is spread and transmitted, and how to interrupt its transmission cycle. This review focuses on understanding cryptosporidiosis, including its infective stage, pathogenesis, life cycle, genomics, epidemiology, previous outbreaks, source of the infection, transmission dynamics, host spectrum, risk factors and high-risk groups, the disease in animals and humans, diagnosis, treatment and control, and the prospect of an effective anti-*Cryptosporidium* vaccine. It also focuses on the role of the One Health approach in managing cryptosporidiosis at the animal–human–environmental interface. The summarized data in this review will help to tackle future *Cryptosporidium* infections in humans and animals and reduce the disease occurrence.

## 1. Introduction

Cryptosporidiosis is an enteric disease caused by a protozoon parasite belonging to the genus *Cryptosporidium*. It is one of the most prevalent waterborne diseases and the leading cause of waterborne disease outbreaks worldwide [1,2,3]. More than 58 million cases of diarrhea are detected annually in children and are associated with protozoal infections. Specifically, waterborne pathogens such as *Cryptosporidium* and *Giardia* were involved in the World Health Organization’s “Neglected Disease Initiative” [4,5,6]. Although *Cryptosporidium* infections are acute self-limiting gastroenteritis in immunocompetent individuals, chronic and life-threatening diarrheal disease can develop in immunocompromised individuals. Neonates are highly susceptible to infections due to their immature immune system, and they can become infected by ingestion of low doses of the parasite’s oocysts. Annually, diarrheal diseases have caused up to 1.6 million deaths worldwide. One-third of these deaths have been reported in children under 5 years due to contaminated drinking water and poor hygiene [7]. *Cryptosporidium* causes up to 20% of all cases of diarrhea in children in developing countries and causes fatal complications in HIV-infected persons [8]. *Cryptosporidium* is also responsible for more than 8 million foodborne illness cases worldwide annually [9]. Cryptosporidiosis primarily affects people who are living in rural and in urban slums, where there is a high probability of disease transmission and spread [10]. 

The human medical importance of *Cryptosporidium* was highlighted in 1982, after the CDC report on *Cryptosporidium*-induced diarrheas in patients infected with Human Immunodeficiency Virus (HIV). The international interest in *Cryptosporidium* as a public health problem began in 1993 after the largest global waterborne outbreak, when more than 400,000 inhabitants in Milwaukee, Wisconsin, USA were infected with *C. hominis* due to the consumption of contaminated drinking water [11,12,13]. From 2014 to 2016, the center for disease control (CDC) in the USA reported a doubled increase in the number of *Cryptosporidium*-associated waterborne infections [14] with an estimated 748,000 annual human cases [15]. In addition, the risk of cryptosporidiosis is increased in developing countries due to poor water and food sanitation [16]. In developing countries, children under five years old are the most affected groups with *Cryptosporidium* [17]. The oocysts can survive outside the host for several months and retain infectivity, despite adverse environmental conditions such as salinity and the presence of chemicals [18,19,20]. Mixed infections in calves with *Cryptosporidium*, enterotoxin *Escherichia coli* (ETEC) as well as the corona- and rotaviruses, are considered the most important reason for the calf diarrhea complex [21]. To date, there are no effective chemotherapeutics for the treatment of cryptosporidiosis [22,23]. Nitazoxanide and halofuginone in humans and animals are the approved drugs against *Cryptosporidium* infection. However, their application does not guarantee treatment efficacy [24,25,26]. Therefore, the control of cryptosporidiosis should be based mainly on (1) reducing the prevalence of infection, (2) breaking the transmission pathways between animals and humans, and (3) maintaining a good hygienic environment for humans and animals. Information about the route and spread of *Cryptosporidium*, the magnitude of infections, and the major sub-species prevailing in animals and humans, is important to achieve effective control. This epidemiological information, in addition to the One Health approach, will help to initiate planning for the control of cryptosporidiosis.

## 2. Life Cycle and Developmental Stages of *Cryptosporidium*

**Figure 1:**
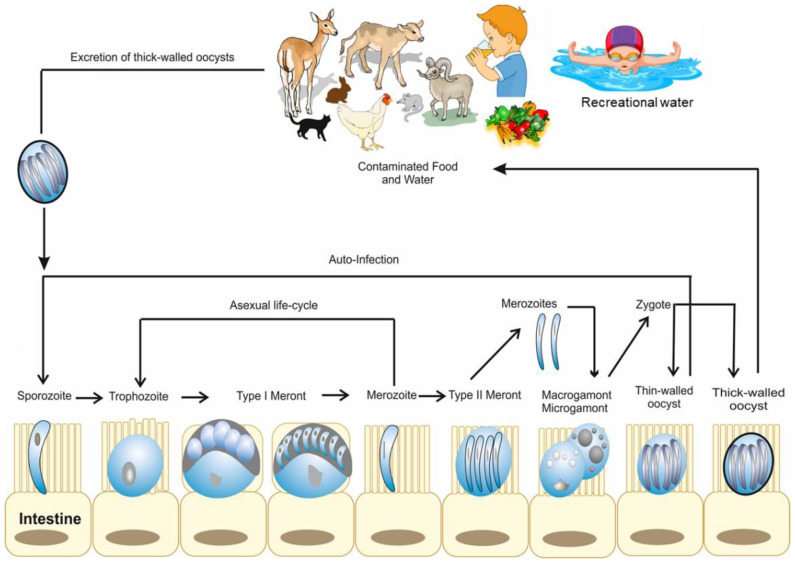
Life cycle and developmental stages of *Cryptosporidium* in animals and humans [46].

*Cryptosporidium* belongs to the Coccidia class of the phylum Apicomplexa. *Cryptosporidium* have some features which differentiate them from all other Coccidia [27], including (1) intracellular and extra-cytoplasmic localization, (2) forming of a “feeder” organ, (3) presence of morphological (thin- or thick-walled) oocysts as well as functional (auto vs. new-infection) types of oocysts, (4) small size of oocysts, (5) missing some morphological characteristics such as sporocysts or micropyles, and (6) the resistance of *Cryptosporidium* to all the available anti-coccidial drugs [27,28]. *Cryptosporidium* has a complex monoxenous life cycle, which is divided into two phases: the asexual phase (sporogony and schizogony/merogony) and the sexual (gamogony) phase. They proliferate and differentiate during the invasion of the free-living stages of *Cryptosporidium* within the parasitophorous vacuole under the brush border of the host cell located outside the cellular cytoplasm [29]. *Cryptosporidium* parasites can then attach to the cell surface and move along it for a short time using gliding mobility before they start to enter the cell. *Cryptosporidium* does not completely invade the cells actively, but they provoke the cells to embrace them with a host-cell-derived membrane. Additionally, at the parasite–cell interaction phase, the *Cryptosporidium* creates an actin-rich disk, a feeder organelle responsible for nutrition intake, as well as a channel into the cytoplasm of the host cell [30]. After *Cryptosporidium* internalization in the host cells, the sporozoite divides inside the parasitophorous vacuole to approximately 4 µm × 4 µm in diameter as a spherical trophozoite with an excentric cell nucleus. After three asexual divisions (merogony/schizogony), the trophozoite is divided into 5 µm × 5 µm large type-1 meront, which contains eight merozoites. The merozoites and the sporozoites are similar in shape and size; however, the nucleus of the merozoites is located more centrally to the cell compared to the sporozoites. Upon leaving the parasitophorous vacuole, the merozoites begin their asexual development cycle in the epithelial cells and develop Type-I meronts again, then the trophozoite. Otherwise, the merozoites initiate the sexual development cycle through differentiation to type-II meronts. Inside the meront, four merozoites develop by asexual division and after infection of further enterocytes, they are divided into micro- and macro-gametes (gamogony). The immature micro-gamontes are spherical, 5 µm × 4.5 µm in diameter, contain up to 16 peripherally located compact cell nuclei, and are precursors of the developing micro-gametes (Figure 1). They also have stubbed front ends and cell nuclei with no flagella. The mature micro-gametes leave their host cell and fertilize the macrogametes. Macrogametes are spherical, 5 µm × 5 µm in diameter and contain granulated cytoplasm and eccentrically positioned wall-forming bodies. Tandel et al. have suggested the direct development of gametes from type I meronts [31]. The zygote grows by syngamy and then goes through sporogony-a meiosis-like process. The oocysts (thin- or thick-walled) with 4 haploid sporozoites (sporulated oocysts) develop inside the parasitophorous vacuole (Figure 1) [30,32]. Thin-walled oocysts (about 20%) excystate in the host intestinal tract, leading to endogenous autoinfection, and the thick-walled oocysts (about 80%) are extremely resistant to several disinfectants, are excreted with the feces to the environment and can survive outside the host for a long time [33]. The thick-walled oocysts represent the exogenous stage of the *Cryptosporidium* parasite. *Cryptosporidium* oocysts are approximately 4µm× 6µm in diameter, spheric to ovoid shape, have a residual body, and four banana-like or comma-shaped sporozoites with a pointed front end and a stubbed hind end, where the nucleus is localized [34,35,36]. The residual bodies are 2.4 µm × 2.5 µm in diameter and consist of a spherical to ovoid membrane-bound globule (1.5 µm × 1.6 µm) and are surrounded by small granules (0.2 µm × 1.2 µm). *Cryptosporidium* sporozoites are not encapsulated by a sporocyst and the oocyst wall consists of an outer and an inner layer, and a pre-formed junction that extends from one pole of the oocyst to approximately half of the oocyst [34]. Additionally, four sporozoites (5µm× 1µm in diameter) hatch out of the pre-formed joint under the effect of temperature, pH, gall bladder salts, pancreas enzymes, and CO_2_ of the host gastrointestinal tract. The free sporozoites adhere to the microvilli of the enterocytes and lead to internalization using their proximal end. The sporozoites’ glycoproteins (GP40 and GP900 of 40 kDa and ˃900 kDa) and the circumsporozoite-like glycoprotein (CSL) play an important role in the adhesion and invasion process of the sporozoites to the host cells [32,37]. The host cell surrounds the sporozoites with membrane protrusions and forms a parasitophorous vacuole in the brush border of the enterocyte. Interestingly, the localization of the parasitophorous vacuole by *Cryptosporidium* spp. is different from that of the other Apicomplexa; thus, *Cryptosporidium* spp. localization is described as intracellular, but extracytoplasmic [38]. Additionally, the feeder organelle develops at the sporozoite and host cell membrane contact point. They supply the maturing parasite with nutrients and facilitate internalization [39]. The molecular components and mechanisms involved in the *Cryptosporidium* development cycle have previously been described [30]. 

The infectious stage (sporulated oocyst) of *Cryptosporidium* was reported to be excreted in large numbers in the feces of experimentally infected calves (up to 4 × 10^7^ oocysts per gram of feces) [40], or excreted with the bronchial exudates in the case of respiratory cryptosporidiosis and which immediately contaminated the environment [41]. The sporulated oocysts are very resistant to environmental factors and only a few chemical disinfectants show efficacy against the sporulated oocysts due to their thick wall [42]. Therefore, it is difficult to completely remove the *Cryptosporidium* oocysts from contaminated drinking water [43]. The thick wall oocysts are sporulated and are infectious when shedding, which can result in immediate infection of new hosts. The infectious dose of *Cryptosporidium* oocysts for humans is about nine oocysts per *Cryptosporidium* isolate and about 50 oocysts for calves [44,45]. However, it was reported that 1 to 10 oocysts of *Cryptosporidium* caused infection for some individuals during the Milwaukee outbreak [42]. Although, one infected host can shed up to 10^10^ oocysts, which results in a huge infection pressure.

## 3. Pathogenesis of *Cryptosporidium*

After ingestion of the thick-walled oocyst with food or water by the host, many signaling molecules are expressed on the sporozoite surface that mediate their attachment and invasion to the host cells. Calcium-dependent protein kinases (CDPKs) were reported to be involved in the regulation of the invasion process of the sporozoite to the host cell [30,47]. Furthermore, *Cryptosporidium* is embraced by the host cell instead of invading the host cells. Therefore, it stays in an epicellular location and this induces tremendous actin rearrangement in the infected cells [30]. After attachment and invasion of *Cryptosporidium,* the host–parasite interactions play an important role in pathogenesis [48]. In calves, *C. parvum* causes acute to chronic catarrhal enteritis that begins in the distal ileum; however, different *Cryptosporidium* developmental stages were also detected in the duodenum, colon, and part of the cecum. The affected mucosa is hyperemic and edematous and the mesenteric lymph nodes are partially enlarged and edematous [49]. Histologically, mild to moderate villus atrophy associated with occasional villus fusion was observed. The affected crypts are partially dilated and contain neutrophil granulocytes. The lamina propria mucosa also had neutrophil granulocytes and a large mononuclear cell infiltration [50]. In the infected host, epithelial cell degeneration, metaplasia of physiological high prismatic to isoprismatic villus epithelial cells, hyperplastic crypt epithelium, displacement of microvilli in the area of the intracellular parasite stages’ attachment zone, and long microvilli can be seen in the vicinity of the parasite stage [51]. These pathological alterations result in the reduction of the intestinal absorption surface and, consequently, malabsorption. Damage to the intestinal epithelium may also have an impact on the activity of brush border membrane enzymes (glucoamylase, alpha-dextrinase, saccharase, lactase), resulting in a reduction in the small intestine’s carbohydrate digestion ability. As a result, osmotically active particles persist in the intestinal lumen, osmotic diarrhea develops, and water resorption is impeded. Several causes can lead to increased chloride secretion into the gut lumen, including immune response to membrane injury, prostaglandins secreted by enterocytes of intra- and sub-epithelial lymphocytes, and plasma cells and macrophages that enhance blood vessel permeability [50]. 

## 4. Species, Genotypes/Subtypes, and Host Spectrum of *Cryptosporidium*

Currently, there are more than 40 morphologically and molecular-biologically different *Cryptosporidium* species [52,53,54,55,56], which infect mammals (Bovidae, Primates, Carnivora, Hares, Equidae, Rabbits, Rhinocerotidae, and Tapiridae), amphibians, birds, and reptiles. Additionally, more than 157 mammalian species were listed as hosts for *Cryptosporidium* infection [57]. However, *Cryptosporidium* species including *C. hominis, C. bovis, C. parvum, C. ryanae*, *C. andersoni*, *C. fayeri*, *C. canis*, *C. felis*, *C. macropodum*, *C. muris*, *C. suis*, and *C. wrairi* have been isolated from mammals. *C. meleagridis*, *C. baileyi*, and *C. galli* have been isolated from birds [58], while *C. varanii* and *C. serpentis* have been isolated from reptiles and *C. fragile* has been isolated from amphibians (Table 1) [59]. Additionally, *C. rubeyi* has been isolated from squirrels, *C. scophthalmi* from turbot, *C. huwi* from fish, and *C. erinacei* from horses and hedgehogs [60]. Human cryptosporidiosis is caused by *C. hominis*, while *C. parvum* is considered the zoonotic species of human cryptosporidiosis [61]. Both *C. hominis* and *C. parvum* are responsible for more than 90% of human cryptosporidiosis. Although there is host specificity of the *Cryptosporidium* species, other species such as *C. meleagridis*, *C. baileyi*, *C. andersoni*, *C. canis*, *C. felis*, *C. bovis*, *C. suis*, *C. fayeri*, *C. scrofarum*, *C. tyzzeri*, *C. erinacei*, and *C. muris* have been detected in animal hosts as well as in humans. The aforementioned species and *C. parvum* have been considered potentially zoonotic species [62,63]. Additionally, humans can also be infected with *C. viatorum*, *C. cuniculus*, *C. ubiquitum*, Chipmunk genotype I, *Cryptosporidium* horse, and *Cryptosporidium* mink genotype (Table 1) [64]. 

Currently, there are more than 60 reported genotypes of *Cryptosporidium* that differ in their molecular sequences [56,65]. *Cryptosporidium* subtypes are distinguished by the number of repeats in each strand. Short, repetitive sequences (R) appear directly after the trinucleotide repeats in some subtypes. In *C. parvum*, 11 subtype families (IIa- IIk) have been discovered with at least 78 subtypes. Furthermore, in *C. hominis*, six subtype families have been detected (Ia, Ib, Id, Ie, If, and Ig) with at least 78 subtypes [63,66,67,68]. In *C. meleagridis*, seven subtype families have been identified (IIIa- IIIg), while six subtype families were identified in *C. fayeri* (IVa- IVf), and two in *C. cuniculus* (Va, Vb), Horse genotype (VIa, VIb), and *C. tyzzeri* (IXa, IXb), whereas one subtype was identified in *C. erinacei* (XIIIa), Mink genotype (Xa), Ferret genotype (VIIIa), and *C. wrairi* (VIIa) [63]. Several highly preserved genes, including (1) small subunit rRNA (18S rRNA), (2) *Cryptosporidium* oocyst wall protein (COWP), (3) heat shock protein (HSP70), and (4) the actin gene, can differentiate between *C. parvum* and *C. hominis*. The 18S rRNA gene is crucial because it contains multiple conserved regions within the *Cryptosporidium* genus. This makes primer development that targets most *Cryptosporidium* species easier. The amplification of the extracted DNA from the oocysts can be performed using conventional or nested polymerase chain reaction (nPCR). It is difficult to identify the mixed infections of distinct *Cryptosporidium* genotypes by using PCR with 18S rRNA, COWP, HSP70, and the actin gene. On the other hand, the GP60 gene is advantageous because the species with the highest affinity for the primer (species-specific) will be amplified to a greater extent than the others, allowing the dominant species to be identified alone [69]. Additionally, the 5’ end of the GP60 gene has a highly variable area of microsatellites, which consists of trinucleotide repeats (TCA, TCG, TCT), which all code for the amino acid serine. Amplicon next-generation sequencing (NGS), which can identify low-abundance sequences in mixed infections, has shown that it can identify additional *Cryptosporidium* gp60 subtypes in various hosts that were not identified by Sanger sequencing [70,71]. This has important implications for tracing the zoonotic transmission of *Cryptosporidium*, as Sanger sequencing may not detect zoonotic species and subtypes that are present at low abundance and therefore incorrect conclusions regarding zoonotic transmission may be made [72].

**Table 1:** Most predominant *Cryptosporidium* species: major hosts, oocyst sizes and locations [18,58,73,74,75,76].

*Cryptosporidium* spp.	Hosts	Sporulated Oocyst Size (µm)	Location
*C. hominis*	Humans	4.5 × 5.5	Small intestine
*C. parvum*	Ruminants, humans, deer	4.5 × 5.5	Small intestine
*C. bovis*	Ruminants	4.2–4.8 × 4.8–5.4	Small intestine
*C. andersoni*	Ruminants, camel	5.5 × 7.4	Abomasum
*C. ryanae*	Ruminants	3.2 × 3.7	Small intestine
*C. xiaoi*	Sheep	3.9 × 3.4	Small intestine
*C. ubiquitum*	Sheep/wildlife	5.2 × 4.9	Small intestine
*C. meleagridis*	Chicken, turkey, humans	4.5–5.0 × 4.6–5.2	Intestine
*C. baileyi*	Birds	6.4 × 6.2	Cloaca, bursa, respiratory tract
*C. galli*	Birds	8.0–8.5 × 6.2–6.4	Proventriculus
*C. avium*	Birds	5.3–6.9 × 4.3–5.5	Intestine
*C. ornithophilusis*	Ostrich	6.13 × 5.15	Intestine
*C. proventriculi*	Psittaciformes birds	7.4 × 5.8	Proventriculus
Avian genotype II	Birds	6.0–6.5 × 4.8–6.6	Intestine
Avian genotype IV	Birds	8.25 × 6.3	Intestine
Eurasian woodcock genotype	Birds	8.5 × 6.4	Intestine
*C. suis*	Pigs, humans	5.1 × 4.4	Small intestine
*C. wrairi*	Guinea pigs	4.0–5.0 × 4.8–5.6	Small intestine
*C. cuniculus*	Rabbits	5.9 × 5.4	Small intestine
*C. canis*	Canids, humans, mink, fox, coyote	5.0 × 4.7	Small intestine
*C. felis*	Felids, humans	4.5 × 5.0	Small intestine
*C. saurophilum*	Lizards, snakes	4.2–5.2 × 4.4–5.6	Intestinal and cloacal mucosa
*C. serpentis*	Snakes, lizards	4.8–5.6 × 5.6–6.6	Stomach
*C. fayeri*	Red Kangaroo, marsupials	4.9 × 4.3	Intestine
*C. macropodum*	Marsupials	4.9 × 5.4	Small intestine
*C. muris*	Rodents, humans	5.6 × 7.4	Stomach
*C. ratti*	Rodents	4.5–5.4 × 4.5–5.0	Small intestine
*C. tyzzeri*	Mice	4.6 × 4.2	Small intestine
*C. molnari*	Fish	4.7 × 4.5	Stomach
*C. scophithalmi*	Fish	3.0–4.7 × 3.7–5.0	Intestine
*C. nasorum*	Fish	4.3 × 3.2	Intestine

## 5. Epidemiology of Cryptosporidiosis 

### 5.1. Source of Infection and Mode of Transmission

#### 5.1.1. In Humans

The zoonotic transmission of *Cryptosporidium* can take place via direct contact with an infected person and/or consumption of contaminated drinking water or food and/or inhalation of oocysts from contaminated air with aerosolized droplets or fomites [41,77]. Additionally, synanthropic flies (suborder: Cyclorapha) play a crucial role in the mechanical transmission and spread of infection [78]. There are multiple factors leading to human cryptosporidiosis [61] and the occurrence of outbreaks, such as (1) contaminated drinking water, and unclean recreational/swimming pool water, (2) contaminated foods such as raw fruits and vegetables that were fertilized with contaminated effluent, (3) contact with infected people (hospitals, daycare centers, schools), (4) contact with infected animals (especially calves), and (5) anal sexual contact [42]. Even though cryptosporidiosis is primarily a water-based illness, the risk of foodborne transmission is well known. Food contamination with *Cryptosporidium* oocysts can occur during food (vegetables, fruits, seafood, and meat) manufacturing, processing, and preparation. The oocysts’ resistance can help them survive various processing procedures, such as chlorine baths and blast freezing [79]. Furthermore, washing fresh fruit may not be enough to eliminate contaminated oocysts, which not only stick to surfaces but can also permeate leafy vegetables through stomatal pores [80,81]. There have been fewer reported foodborne cryptosporidiosis outbreaks than waterborne infections.

#### 5.1.2. In Animals

Calves usually become infected with cryptosporidiosis by ingestion of oocysts from the contaminated environment. There are many possible sources of infection including (1) shedding of infected neighbor animals, (2) contaminated stables, (3) dirty udders and teats of cows, and (4) contaminated water. The subclinical infected adult cattle act as oocysts shedders [82,83], therefore they are considered a potential reservoir for infection. Furthermore, *Cryptosporidium* infection can be also transmitted by animal handling personnel through dirty shoes and clothes as well as via infected dogs, cats, rodents, wild animals, insects (flies, cockroaches, and beetles), and free-living amoeba [84,85]. Mixed infection of *Cryptosporidium* together with enterotoxin *E. coli*, Corona- and Rotaviruses is considered one of the most common causes of neonatal calf diarrhea. The prevalence of bovine cryptosporidiosis ranged between zero and 100% and the prevalence tends to decrease with the increasing age of the animal [86]. There is variation in the tendency of *Cryptosporidium* species to infect calves in an age-dependent manner. For example, *C. parvum* is the most prevalent species in calves up to 8 weeks old, while *C. bovis* is dominant in calves ranging between 2 to 11 months of age [87,88].

### 5.2. Clinical Signs and High-Risk Groups of Cryptosporidiosis 

#### 5.2.1. In Humans

Many risk factors are implicated in the zoonotic transmission of *Cryptosporidium* infection. These factors include contact with infected animals, age (infection rate is higher in young animals and humans), gender (infection is higher in males compared to females), poverty, overcrowding, season (rise of cases around rainy season), poor water quality, poor hygiene measures, the status of the host immunity, exposure to HIV–infected people [89], and natural disasters (storms, earth erosions, floods) [90]. The high-risk groups of people that can be exposed to *Cryptosporidium* infection include: (1) children in childcare centers, (2) childcare workers who change children’s diapers, (3) parents or attendees of infected children, (4) the elderly (75 years and older), (5) travelers to/from endemic areas, (6) swimmers who swallow contaminated water, (7) people handling infected animals and birds, (8) people who have been sexually exposed to human feces, (9) people taking care of other people who are infected, (10) people who drink from untreated water such as backpackers, hikers, and campers [91], (11) organ transplant recipients, and (12) other occupational associated groups such as veterinarians, animal handlers (sweepers, vaccinators, debeaking staff), pet owners, and hunters. 

The severity of clinical signs in infected humans depends on the age and the immunity of the infected person [92,93]. The incubation period in immunocompetent people is from 5 to 21 days, followed by acute self-limiting diarrhea that lasts 3 to 12 days. The clinical signs range from medium to profuse watery to catarrhal diarrhea, which is often associated with abdominal pain, nausea, vomiting, flatulence, fatigue, and anorexia. Respiratory symptoms such as cough, sneezing, and expectoration may occur after inhalation of oocysts from contaminated air [41,56]. Asymptomatic infection can also occur [94,95]. However, the infection can develop into a chronic and life-threatening disease in immunocompromised persons [95], specifically people suffering a genetic immunological malfunction such as hyper-IgM syndrome, a significant reduction in the number of CD4-lymphocytes such as HIV infection, or those undergoing immunosuppressive therapy after organ transplantation [96]. *Cryptosporidium* has been isolated from the gallbladder and the respiratory tract of HIV/AIDS patients as well as from patients suffering from severe combined immune deficiencies (SCID), causing cell-mediated immunity deficiency, and extra-intestinal forms (in the ductus pancreaticus a.o. and the respiratory bronchioles) [96]. Differences in clinical symptoms have been noted between *C. parvum* and *C. hominis* in children and HIV/AIDS patients, with *C. parvum* being less virulent than *C. hominis* [97,98]. In HIV patients, *C. parvum* infections are mostly associated with vomiting and chronic diarrhea and are more frequent than *C. hominis* infections [97]. Additionally, *Cryptosporidium* infection at a young age has been linked to stunted growth and long-term cognitive problems, particularly in children in developing countries [42].

#### 5.2.2. In Livestock Animals 

Cryptosporidiosis is more frequent in young calves and the severity of the disease depends on various factors including age, infectious dose, immunity of the host, season, geographical distribution, and mixed infection with other pathogens [99]. The clinical signs vary from asymptomatic to pasty or watery profuse diarrhea, dehydration, and death. Co-infections of *C. parvum* with enterotoxin *E. coli*, Coronaviruses, and Rotaviruses can occur within the first three weeks of age and are considered one of the major causes of mortality in calves [1,94]. Neonatal diarrhea with a single or mixed *C. parvum* infection is characterized by yellowish, profuse diarrhea and is associated with complications such as exsiccosis, metabolic acidosis, and loss of electrolytes [50,100]. Consequently, cryptosporidiosis results in severe economic losses due to morbidity, growth retardation, and treatment costs [101,102]. The prevalence of *Cryptosporidium* in animals varies according to the geographical area, animal species, rearing forms, and the diagnostic tests. For example, the prevalence reached up to 100% in goats and horses in South America and sheep in Europe. Additionally, the highest prevalence of infection in buffalo was reported in Africa (52.0%) and Asia (50%) compared to other continents. The highest prevalence (more than 50%) in cattle was reported in all the continents except South America. The highest prevalence in pigs was reported in Asia (55.8%) (Table 2).

#### 5.2.3. In Poultry

Several *Cryptosporidium* species can infect birds, including *C. meleagridis*, *C. galli*, and *C. baileyi* [58]. These species have different predilection sites. For example, *C. meleagridis* and *C. baileyi* can develop in the small and large intestines as well as the bursa of Fabricius, causing different degrees of enteritis. *C. galli* was reported to infect finches, chickens, and grosbeaks and infect only the proventriculus, while *C. meleagridis* was reported to infect turkeys and parrots. *C. baileyi* is the most common avian *Cryptosporidium* that can infect chickens, turkeys, cockatiels, quails, ostriches, and ducks [105]. *Cryptosporidium* species can also multiply in the tissues of the respiratory tract of the infected birds. Additionally, it causes enteritis and renal disease, due to inflammation of Fabricius’ bursa and kidneys [105,106]. There are approximately 11 *Cryptosporidium* genotypes that have been detected from more than 30 bird species, including avian I–V, duck genotype, goose genotypes I–IV, and the Eurasian Woodcock genotype [76]. However, *Cryptosporidium* avian genotype III has been associated with chronic vomiting in peach-faced lovebirds (*Agapornis roseicollis*) [107]. *Cryptosporidium* species such as *C. hominis*, *C. parvum*, and muskrat genotype have also been isolated from Canada geese (*Branta canadensis*) [108,109,110]. Recently, *C. ornithophilusis* was isolated from farmed ostrich in the Czech Republic [74], while *C. avium* was isolated from red-crowned parakeets [75]. The prevalence of *Cryptosporidium* has been investigated in different species of poultry worldwide. The prevalence ranged between 0.8% in pigeons to 50% in broilers and layers (Table 3). The most detected *Cryptosporidium* species were *C. baileyi*, *C. meleagridis*, *C. galli*, and *C. parvum.* In some countries, scientists have also been able to isolate other species such as *C. avium* from China, *C. muris* from China and Australia, and *C. andersoni* from Australia (Table 3).

### 5.3. Outbreaks of Cryptosporidiosis in Humans 

The first waterborne cryptosporidiosis outbreak was reported in 1993 in Milwaukee, Wisconsin (USA), with an estimated 403,000 people affected, 4400 hospitalizations, and more than 100 deaths [11,12,13]. The CDC reported a doubled increase in the number of *Cryptosporidium*-associated waterborne outbreaks from 2014 to 2017 [14]. Between 2009 and 2017, there were more than 444 reported outbreaks in the USA [2]. The number of outbreaks reported has increased by an average of 13% annually. These outbreaks have resulted in 7465 infected cases with 287 hospitalizations and 1 death. Out of these outbreaks, 156 outbreaks resulted in 4232 cases and 183 hospitalizations and were associated with exposure to *Cryptosporidium* in pools or waterparks. Among these outbreaks, 14.6% were linked to contact with cattle, and 12.8% were linked to contact with infected persons in childcare settings. Among the 22 foodborne outbreaks, 40.9% were linked to unpasteurized milk and 18.2% were linked to unpasteurized apple cider. However, the mode of transmission was unknown for 14.2% of the outbreaks [2]. Interestingly, salad consumption was incriminated in 35% of cases [9]. Between 2010 and 2020, most of the waterborne outbreaks were caused by *C. hominis* (72%), while the majority of foodborne outbreaks were caused by *C. parvum* (96.5%; Table 4) worldwide [135]. Interestingly, most of the reported waterborne outbreaks were linked to swimming pools, whereas most foodborne outbreaks were linked to unpasteurized raw milk and eating salad. During these outbreaks, the most predominant identified *C. hominis* subtype was IfA12G1 in the USA, IbA10G2 in the UK, Sweden, and Australia, and IbA9G2 in French Guiana and Germany. Over the last 10 years, *C. hominis* subtype IfA12G1 was responsible for approximately 50% of *C. hominis*-related waterborne outbreaks in the USA. Furthermore, the most predominant identified *C. parvum* subtype was IIaA15G2R1 in the USA and UK, and IIaA19G1R1 in Norway (Table 4). The majority (64.3%) of foodborne outbreaks caused by *C. parvum* were due to IIa and only 35.7% were due to IId subtypes, which are common in livestock, suggesting its important role in foodborne outbreaks [136].

## 6. Diagnosis of *Cryptosporidium*

There are several methods used for the detection of *Cryptosporidium* directly in fecal samples, including microscopy detection of the oocysts either by using flotation or sedimentation techniques to determine the number of oocysts in the stool [33]. The oocyst detection limit using a microscope has been recorded as low as 50,000 to 500,000 oocysts per gram of feces. Direct detection of *Cryptosporidium* oocysts is usually done by microscopy without any staining and/or by the modified Ziehl–Neelsen stain, where the oocysts are stained purple with a blue background. Fecal smears can be also tested microscopically after staining with the Heine technique or Kinyoun’s Carbol fuchsin staining technique [157]. Additionally, the immunofluorescent antibody-based (IFA) staining techniques using monoclonal antibodies against the oocyst wall antigen are also widely used. These are characterized by high sensitivity and are cheaper compared to other traditional staining methods [73]. In general, the parasitological methods for *Cryptosporidium* detection do not differentiate between viable and non-viable oocysts.

Serological methods are considered the best tools for the screening of large numbers of samples, particularly in epidemiological surveys. The serological tests include enzyme-linked immunosorbent assays (ELISA) and enzyme-linked immunoelectron transfer blots (EITB; Western blot). The enzyme immunoassay (EIA) methods have many advantages as they are faster, easy to perform, inexpensive, and more sensitive compared to the immunofluorescence methods [33]. Rapid immunochromatographic (strip) tests can also be used [158,159] as they are used for the detection of the oocyst cell wall proteins using monoclonal antibodies [160].

The molecular diagnosis of *Cryptosporidium* using nucleic acid detection techniques can differentiate between viable and non-viable oocysts [161]. They can also identify species, genotypes, and subtypes, which is crucial for detecting *Cryptosporidium* prevalence and transmission routes. [162]. Molecular methods include random amplified polymorphic DNA PCR (RAPD-PCR), single-round and nested PCR, reverse transcription PCR (RT-PCR), arbitrary primed PCR (AP-PCR), single-strand conformation polymorphism (SSCP) analysis, crypto PMA-PCR, real-time PCR followed by restriction fragment length polymorphism (RFLP) analysis, melting curve analysis, microarray, and DNA sequencing [163]. These PCR-based methods are more sensitive than conventional microscopical and serological methods and are considered a gold standard [158]. Molecular techniques are very popular as they are used for the differentiation and genotyping of *C. parvum* and *C. hominis* [164]. Molecular diagnosis can detect the target genes of *Cryptosporidium* such as 18S rRNA, COWP, HSP70, and the actin gene. The 18S rRNA gene-specific PCR is extremely useful for detecting a conserved area in the gene or distinguishing between *Cryptosporidium* spp. (targeted nucleotide segments with varied nucleotide sequences) [33,165]. Furthermore, restriction enzymes are employed to differentiate species by digesting amplicons into fragments of varying sizes based on the species, causing the products to migrate at different distances on the gel [166]. Gene sequencing can also be used to identify various *Cryptosporidium* species by using pure DNA that has been amplified using internal primers and tagged with colored nucleotide bases that emit light at different wavelengths [167]. Using the Basic Local Alignment Search Tool, the generated forward and reverse sequences can be assembled into contigs and compared to sequences deposited in the Gene Bank. The dominant species or species with a strong affinity for the primers will be amplified to a greater extent than others, making it difficult to identify the mixed infection using PCR. At gene sequencing, amplification of more than one species manifests itself as multiple peaks in many sites and has difficulty assembling contigs. A combination of various species/genotype-specific primers or cloning of single amplicons produced in the area is required for the successful analysis of mixed infection [168]. Another possibility is to use species-specific primers to undertake GP60 subtype analysis. This GP60 gene is targeted for neutralizing antibodies and is expressed on the apical surface of invading stages (sporozoites and merozoites) [169]. GP60 subtyping can also help in the determination of virulence of different *C. parvum* and *C. hominis* subtypes [170].

## 7. Prevention and Treatment of *Cryptosporidium* Infection

### 7.1. One Health Approach for the Control of Cryptosporidiosis

The “One Health” approach is a worldwide strategy that is used to mitigate zoonotic diseases and improve health by preventing infection occurrence at the human–animal–environment interface. Collaboration between all health sectors (veterinarians, occupational health physicians, and public health operators) can help in infection control by enhancing the educational system, status of thinking, legislation, and administrative structures [171]. The One Health approach has been previously proposed to tackle cryptosporidiosis as well as other zoonotic diseases [171,172,173], since there is a critical need for close One-Health-oriented interactions among professionals working in diverse fields such as physicians, veterinarians, diagnosticians, epidemiologists, public health experts, ecologists, economists, social scientists, governments, decision-makers, and pharmaceutical industries. In this review, we propose using the One Health approach as prophylactic prevention for *Cryptosporidium* infection in humans, animals, and the environment through understanding the disease pathogenesis, life cycle, genomics, epidemiology, previous outbreaks, source and transmission dynamics, host spectrum, risk factors, high-risk groups, disease in animals and humans, diagnosis, treatment and control, and the prospect of effective anti-*Cryptosporidium* vaccines. The One Health approach includes (1) increasing public health awareness about cryptosporidiosis and its ways of transmission, (2) breaking the parasite’s transmission cycle, (3) epidemiological investigations to identify risk factors, (4) establishing regular surveillance, (5) treating the infected animals to decrease outbreaks in humans, and (6) training the medical and veterinary specialists on the management and diagnosis of the disease and hiring of professional, well-trained personnel.

### 7.2. Preventive Measures for Cryptosporidium Infection

Due to the absence of effective treatment, the prevention of cryptosporidiosis relies mainly on the elimination and/or reduction of contamination of the environment with infectious oocysts [33]. It is recommended to move animals to clean and dry places and disinfect the contaminated areas, however, this is mostly not applicable on farms with a large number of animals. For humans, continuous disinfection of the contaminated areas will reduce person-to-person transmission in institutional and domestic settings. In general, the infectivity of the oocyst and its survival time will be restored at low temperatures (less than 5 °C) and increased by temperatures higher than 15 °C for 3 months [174]. In general, several physical stresses can affect *Cryptosporidium* oocysts including irradiation, heat, cold, pressure, and desiccation [19]. The infectivity of *C. parvum* oocysts at different temperatures is due to the carbohydrate energy reserve of the sporozoites, and the residual bodies including amylopectin (which helps in the excavation process and the host–cell invasion) granules which are used quickly at higher temperatures [33,175]. Increasing the temperature to 64.2 °C or more for 5 min and 72.4 °C for 1 min renders the oocysts non-infectious [176]. Even in the presence of cryoprotectants, *C. parvum* oocysts can survive at −20 °C for prolonged periods, but not at −70 °C or below [176]. However, ultraviolet (UV) irradiation can render *Cryptosporidium* oocysts non-infectious [177]. The most effective disinfectants against *Cryptosporidium* oocysts are those that contain chlorine dioxide, hydrogen peroxide, or ammonia. Although high concentrations and longer exposure to chlorine-, bromine-, and iodine-related compounds can decrease the infectivity of the oocysts, they are limiting their practical applications. Ozone is one of the most effective chemical disinfectants against *Cryptosporidium* and can be used against *Cryptosporidium* oocysts in water [33]. It has also been reported that rotifers, which occupy rivers, lakes, seawater, and ponds, and predacious protozoa, can ingest oocysts of *C. parvum* [178]. Some rotifers were found to discharge oocysts in boluses containing a mixture of other eaten components [179], and therefore they can be used for *Cryptosporidium* oocyst control in water.

### 7.3. Treatment of Cryptosporidium Infection

Several active compounds have been tested for their efficacy against *Cryptosporidium* infections [23]. There are only a few drugs that possess efficacy in vitro [23,180,181,182]. Halofuginone (a bromo-chlorinated quinazoline derivate) is approved for pro- and metaphylactic treatment for animals in Europe. Halofuginone is applied for 7 days at a dose of 100 µg/kg of body weight, starting from the first 24 h after the onset of diarrhea and/or within the first 24–48 h of life as prophylactic. However, symptoms of poisoning include diarrhea, blood in feces, reduction of milk intake, dehydration, exhaustion, and apathy, which can be observed after using a double therapeutic dosage [24]. Furthermore, nitazoxanide (a nitrothiazolylsalicylamide) was approved by the Food and Drug Administration (FDA) for the treatment of cryptosporidiosis in humans ≥ 1 year of age [183]. Nitazoxanide is an oral suspension that is mostly used at a concentration of 100 mg/5 mL for patients ≥ 1 year of age, while tablets at 500 mg for patients ≥ 12 years of age are used for the treatment [91]. Interestingly, approximately 56% of the 71 *Cryptosporidium* outbreaks were associated with drinking contaminated water [10]. Therefore, control of *Cryptosporidium* is a major challenge for water treatment professionals. *Cryptosporidium* oocysts can pass through different types of filters and are not affected by chlorine and chlorine-based disinfectant. Different filtration methods such as direct filtration, conventional filtration, slow-sand filtration, diatomaceous earth filtration, bag filtration cartridge filtration, and membrane filtration are used in the treatment of infected water. The conventional filtration methods using coagulation, flocculation, and sedimentation are capable of the removal of 99% of *Cryptosporidium* [33]. Sand filtration also uses a biological process to remove *Cryptosporidium* oocysts from the water supply. UV irradiation can also affect the infectivity of *Cryptosporidium* oocysts [184,185], suggesting the efficacy of sunlight in the inactivation of oocysts in environmental water reservoirs [73].

### 7.4. Vaccines Development

Currently, there are no available vaccines to control *Cryptosporidium* infection in humans and animals [173]. There is a critical need to develop vaccines, particularly for high-risk groups such as young children, malnourished populations, and immunosuppressed persons. It has been reported that vaccinating mother cows against other diarrhea-causing pathogens such as rotavirus, coronavirus, and *E. coli* may protect against *Cryptosporidium* infection in calves via colostrum, thus helping the calf to resist the infection during the first weeks of age [186]. To develop an effective vaccine, there is a need to understand the host immune response to infection and the host–parasite interactions [187] as well as understand the innate and adaptive host response [188]. However, the nature of these responses is still unknown and needs further investigation [189,190]. Several trials to produce effective vaccines against cryptosporidiosis have been carried out. It was reported that miRNA plays a crucial role in the protection of the host cell against *Cryptosporidium* and the regulation of miRNA expression levels in epithelial cells [191], while mannose-binding lectin (MBL) can protect against cryptosporidiosis, especially in children and immunocompromised persons with MBL deficiency [192,193,194]. Additionally, several antigens such as *gp*15, *cp*15, and *cp*23 are being developed as vaccine candidates. The *gp*15 antigen is substantially conserved between *C. parvum* and *C. hominis*, and there is a significant cross-reactivity between both species [195], while *cp*23 is conserved among *C. parvum* isolates and found in both the sporozoites and merozoites [187]. Using the *cp*15 vaccines to immunize pregnant goats protect offspring [196]. The vaccines provide a transient reduction of *Cryptosporidium* in the stool of vaccinated goats, but they were not fully protected against the infection [197]. Interestingly, the vaccine that contains multiple dominant antigens may enhance protection against the infection. For example, it was reported that *cp*23 plus *cp*15 divalent vaccine prolonged the prepatent period and reduced the shedding of the oocyst compared to vaccination with *cp*23 alone in mice [198]. Furthermore, serum antibodies to both *cp*23 and *gp*15 protected diarrhea in immunocompetent persons infected with *Cryptosporidium* [199,200]. Collectively, the ideal vaccine should (1) provide lifelong immunity in the vaccinated population, (2) protect against species and subtypes of *Cryptosporidium* to assure cross-protection against the most common species infecting humans, and (3) prevent *Cryptosporidium* transmission [187,189]. 

## 8. Conclusions and Future Perspectives

*Cryptosporidium* is one of the water- and foodborne pathogens with socioeconomic and public health importance worldwide. The infection is characterized by high morbidity and high mortality. *Cryptosporidium* infection is ubiquitous and has a high prevalence in animals and humans. Children under 5 years of age and immunocompromised individuals are the most susceptible groups to infections. Cryptosporidiosis in animals has become more common because of environmental contamination in livestock production. *Cryptosporidium* infection can be transmitted directly via drinking/ingestion of contaminated water or food with sporulated oocysts. Most of the foodborne outbreaks associated with *Cryptosporidium* are zoonotic. To prevent disease outbreaks, routine surveillance systems and the application of the One Health approach are required. Food safety and water sanitation are required to prevent and/or reduce future outbreaks worldwide. Each of the available diagnostic tools has its limitations in terms of isolation, detection of co-infections with other pathogens, and cost. In developing countries, the true burden of cryptosporidiosis is underestimated and underreported due to the limitation of diagnostic tools, which results in ineffective clinical and public health management of the disease. Therefore, there is a critical need to develop rapid, reliable, and cost-effective diagnostic tests to improve the detection, reporting, and interpretation of results. *Cryptosporidium* infection prevention and control can be achieved via understanding the sources of the infection (humans and animals), the routes of transmission, the oocyst survival in the environment, and the risk factors. Currently, no effective drugs or vaccines are available to treat and/or prevent infection in animals and humans. There is also a critical need for further studies for the development of effective vaccines. Additionally, more research is needed to develop highly effective disinfection methods for treating Cryptosporidium-contaminated swimming pools and water supplies. 

## Figures and Tables

**Table 2:** *Cryptosporidium* prevalence in livestock animals in different continents [99,103,104].

Continents	Animal Species	Diagnostic Test *	Prevalence Range
South America	Buffalo	CM, PCR	9.4–48.2%
	Cattle	CM, ICT, PCR	3.0–56.1%
	Goat	CM	4.8–100%
	Sheep	CM, PCR	0.0–25.0%
	Pig	CM, PCR	0.0–2.2%
	Horse	CM	0.0–100%
	Calves	CM, ELISA, PCR	84.2%
North America	Cattle	CM, IFA, PCR	1.1–78.0%
	Goat	CM	20.0–72.5%
	Sheep	CM, IFA, PCR	20.0–77.4%
	Pig	CM, IFA	2.8–19.6%
	Horse	CM, IFA, PCR	0.0–17.0%
Africa	Buffalo	CM, PCR	1.3–52.0%
	Cattle	CM, ELISA, PCR	0.5–86.7%
	Goat	CM, ELISA	0.0–76.5%
	Sheep	CM, ELISA, PCR	1.3–41.8%
	Pig	CM, ELISA, IFA, PCR	13.9–44.9%
	Horse	CM, PCR	0.0–2.9%
Asia	Buffalo	CM, ICT, PCR	3.6–50.0%
	Cattle	CM, ICT, IFA, PCR	1.5–93.0%
	Goat	CM, ICT, IFA	0.0–42.9%
	Sheep	CM, ELISA, ICT, PCR	1.8–66.6%
	Pig	CM, IFA, PCR	0.4–55.8%
	Horse	CM, PCR	2.7–37.0%
Europe	Buffalo	ELISA	14.7%
	Cattle	CM, ELISA, ICT, IFA, PCR, QLAT	0.0–71.7%
	Goat	CM, ELISA, IFA	0.0–93.0%
	Sheep	CM, IFA, ELISA	1.4–100%
	Pig	CM, IFA, PCR	0.1–40.9%
	Horse	CM, ELISA, IFA, PCR	3.4–25.0%
Australia	Buffalo	PCR	13.1–30.0%
	Cattle	CM, IFA, PCR	3.6–73.5%
	Goat	PCR	4.4%
	Sheep	PCR	2.2–81.3%
	Pig	CM, PCR	0.3–22.1%

* CM, conventional microscopy; IFA, immunofluorescence antibody test; ELISA, enzyme-linked immunosorbent assay; ICT, immunochromatographic test; QLAT, quantitative latex agglutination; and PCR, polymerase chain reaction. The reported prevalence range was summarized from different research articles.

**Table 3:** Prevalence of *Cryptosporidium* species of birds in different countries.

Country	Species/Genotype	Host	Prevalence	Reference
Brazil	*C. meleagridis*, *C. baileyi*	Chicken, turkey, quail	14.8%	[111]
Brazil	*C. baileyi*, *C. parvum*, *C. meleagridis*	Chickens	12.6%	[112]
China	*C. baileyi*	Chickens	2.4%	[113]
Iraq	*C. baileyi*, *C. parvum*, *C.galli*, *C. meliagredis*	Broilers, layers	50%	[114]
Iraq	*C. parvum and C. baileyi*	Wild pigeons	6.0%	[115]
Iran	*C. parvum and C. baileyi*	Broilers	8.0%	[116]
China	*C. parvum and C. baileyi*	Wild birds	8.9%	[117]
Bangladesh	*C. baileyi*, *C. meleagridis*, *C. parvum*	Layers, broilers, pigeons	15.7%	[118]
China	*C. avium*, *C. baileyi*, *C. galli*, *C. meleagridis*	Chickens	13.7%	[119]
Germany	*C. parvum*, *C. baileyi*	Turkey, broilers, layers	7.0%	[58]
Spain	*C. meleagridis*, *C. parvum*	Wild birds	8.3%	[120]
China	*C. baileyi*, *C. meleagridis*	Pigeons	0.8%	[121]
Czech Republic	*C. baileyi*, *C. meleagridis*	Red-legged partridge	22%	[122]
China	Avian genotype II, *C. baileyi*, *C. meleagridis*	Chickens	9.9%	[123]
China	*C. baileyi*, *C. muris*	Ostrich	10.2%	[123]
Vietnam	Avian genotype II	Ostrich	23.7%	[124]
Algeria	*C. baileyi*, *C. meleagridis*	Broilers	9–69.0%	[125]
Algeria	*C. meleagridis*	Turkey	43.9%	[126]
China	*C. baileyi*, *C. meleagridis*	Japanese quail	13.1%	[127]
China	*C. galli*, *C. meleagridis*, *C. baileyi*, *C. parvum*, Avian genotypes I, II, III, V	Pet birds	8.1%	[128]
Brazil	*C. baileyi*, Avian genotype II, *C. galli*	Wild birds	6.6%	[129]
China	*C. baileyi*	Ostrich	11.7%	[130]
China	*C. baileyi*	Pekin ducks	16.6%	[131]
China	*C. baileyi*, *C. meleagridis*	Chickens	8.9%	[131]
USA	*C. parvum*	Turkey	6.3%	[132]
Brazil	C. *baileyi*, Avian genotypes I, II, III, *C. galli*, *C. meleagridis*, *C. parvum*	Captive birds	4.9%	[133]
Australia	Avian genotypes I, II, III, *C. andersoni*, *C. baileyi*, *C. galli*, *C. muris*	Several avian species	6.3%	[134]

**Table 4:** Recent reported outbreaks of human cryptosporidiosis [135].

Country	Year	No. of Cases	Species/Subtype	Source	No. of Outbreaks	References
**USA**	2017	41	*C. hominis* IfA12G1 and IaA15R3	Swimming pool	3	[137]
	2016	1373	*C. hominis* IbA10G2 and IfA12G1, and *C. parvum* IIaA17G1R1 and IIaA15G2R1	Swimming pool and water park	16	[137]
	2016	10	*C. parvum* IIaA15G2R1 and IIaA18G3R1	Raw cow milk	2	[137,138]
	2015	55	*C. hominis* IfA12G1	Swimming pool	1	[139]
	2015	103	*C. parvum* IIaA17G2R2	Raw milk	1	[137]
	2014	68	*C. hominis* IdA17and IfA12G1	Swimming pool, water slide, and fountain	4	[137]
	2014	11	*C. parvum* IIaA16G3R1	Unpasteurized goat milk	1	[140]
	2013	67	*C. hominis* IaA28R4 and IfA12G1, and *C. parvum* (unknown subtype)	Swimming pool, lake, water park, and fountain	6	[137]
	2013	172	*C. parvum* IIaA15G2R1 and *C. parvum* (unknown subtype)	Drinking water	3	[137]
	2013	21	*C. parvum* IIaA17G2R1 and *C. hominis* (unknown subtype)	Unknown	3	[137]
	2012	182	*C. hominis* IbA10G2, and *C. parvum* IIaA16G3R1, IIaA15G2R1, and IIaA16G2R2	Lake, fountain, water park, and swimming pool	9	[137]
	2011	44	*C. hominis* IaA15R3 and IaA28R4	Water park and swimming pool	2	[137]
	2010	162	*C. hominis* IaA24R4, IaA28R4, IbA10G2 and IdA15G1	Splashpad, lake, water park, and swimming pool	4	[137]
**UK**	2017	43	*C. hominis* IbA10G2 and IbA12G3	Swimming pool	2	[141]
	2016	111	*C. hominis* IbA10G2 and IdA16, and *C. parvum* (unknown subtype)	Swimming pool	10	[141]
	2015	83	*C. hominis* IbA10G2 and IaA14R3, and *C. parvum* IIaA15G2R1 and IIaA26G1R1	Swimming pool and hydrotherapy pool	11	[141]
	2015	424	*C. parvum* IIdA24G1	Salad	1	[135]
	2014	109	*C. hominis* IaA14R3, IaA20R3, IbA10G2 and IdA25, and *C. parvum* IIaA15G2R1 and IIdA17G1	Swimming pool and hydrotherapy pool	11	[141]
	2014	12	*C. parvum* IIaA15G2R1	Drinking water	1	[141]
	2013	94	*C. hominis* IbA10G2 and IA14R3	Swimming pool and paddling pool	5	[141]
	2013	23	*C. hominis* IbA10G2 and IdA18	Public drinking water supply	1	[141]
	2013	11	*C. parvum* IIaA15G1R1	Unpasteurization dairy milk		[141]
	2012	176	*C. hominis* IbA10G2 and *C. hominis* (unknown subtype)	Swimming pool and hydrotherapy pool	10	[141]
	2012	648	*C. parvum* IIaA15G2R1	Pre-cut mixed salad leaves	1	[142]
	2011	21	*C. hominis* IbA10G2 and *C. hominis* (unknown subtype)	Swimming pool	1	[141]
	2010	78	*C. hominis* (unknown subtype)	Swimming pool	2	[143,144]
**Sweden**	2019	122	*C. parvum* IIdA22G1c	Spinach in vegetable juice	1	[145]
	2011	872 + 730	*C. hominis*	Public drinking water source	2	[146]
	2010	27,000	*C. hominis* IbA10G2	Public drinking water source	1	[147]
	2010	16 + 89	*C. parvum* IIdA20G1e and *C. parvum* IIdA24G1	Salad garnish on chanterelle sauce	2	[148]
**French Guiana**	2014	12	*C. hominis* IbA9G2, IbA10G2, IbA15G1	Playing and bathing in a river	1	[149]
**Germany**	2013	167	*C. hominis* IbA9G2	Playing and bathing in a river	1	[150]
**Ireland**	2012	12	*C. parvum* IIaA20G3R1	Public drinking water supply	1	[151]
**Norway**	2018	6	*C. parvum* IIaA14G1R1	Apple juice	1	[152]
	2012	145	*C. parvum* IIaA19G1R1	Goat kids and lambs	1	[153]
**Finland**	2012	>250	*C. parvum* IIdA17G1	Salad	5	[154]
**South Korea**	2012	126	*C. parvum* (unknown subtype)	Tap water from the underground water tank	1	[155]
**Australia**	2012	18	*C. hominis* IbA10G2	Swimming pool	1	[156]
**Canada**	2010	12	*C. hominis* (unknown subtype)	Recreational water park	1	[3]
